# A Lecture on Epistaxis, Vel Hæmorrhagia Nasi

**Published:** 1839-03-02

**Authors:** N. Chapman

**Affiliations:** Professor of the Theory and Practice of Physic, in the University of Pennsylvania


### MEDICAL EXAMINER.

DEVOTED TO MEDICINE, SURGERY, AND THE COLLATERAL SCIENCES.

No. 9.] PHILADELPHIA, SATURDAY, MARCH 2, 1839. [Vol. II.

### A LECTURE ON EPISTAXIS, VEL HÆMORRHAGIA NASI.

*By* N. Chapman, M. D., *Professor of the Theory and Practice of Physic, in the University of Pennsylvania.*

(Concluded from page 118.)

Epistaxis exacts some difference in the treatment.

Connected with a state of vascular force, or cerebral determination, the blood should be permitted to flow as an effort of nature to afford relief, and, when demanded, we have to call to her aid venesection, or local bleeding, and successively, the evacuant and antiphlogistic measures of nearly every description.

To obviate a recurrence of this state of things, the same course may be required to be pursued for a length of time, consisting in occasional bleeding, purging, the liberal use of the nitrate of potash, low vegetable diet, moderate exercise, and in the careful avoidance of the exciting causes.

It has, indeed, been questioned, how far it is proper, under such circumstances, to interfere at at all with a hæmorrhage usually so beneficial. The fact is, that our exertions are intended for the removal of the condition producing it, and which, if allowed to remain, instead of this safe discharge from the nostrils, may occasion a cerebral or some other effusion, where the consequences become alarming, or even fatal.

Epistaxis, however, is, also, of a less active nature, and is then marked by no repletion of the circulation. The pulse is without augmentation of volume, or force, or may be considerably below the natural degree in these respects, or small, quick, and irritated, denoting a debilitated system, and which is further manifested by the pallid, or sallow skin, cold feet, soft and flabby integuments, and loss of muscular power. The blood which escapes is of a light colour, and very thin, as if diluted with water,—yet local congestion may here, sometimes, be detected. Cases of this sort are exceedingly troublesome, recurring very frequently, and sometimes alarmingly copious, and difficult to be restrained.

In the absence of all fulness or excitement, we are called, at once, to suppress the hæmorrhage, as any further expenditure of blood is not allowable, and to effect which a variety of expedients, regular or domestic, have been proposed. The patient is to be placed sitting, in a cool situation, even in a draft of air, with the head inclined backwards, and the feet immersed in a stimulating warm bath. Cold applications, as a cake of ice, or cloths wrung out of the coldest water, are next to be made to the nostrils, or back of the neck, or to the genital organs, which last, having great sensibility, such applications to them prove very effective. Let, at the same time, the sides of the nose be pinched by the fingers, till a coagulum is formed. These means not availing, dossils of lint, dipped into a solution of the sulphate of alumine, or the acetate of lead, or the sulphate of zinc, or copper, or the muriate or sulphate of iron, or the infusion or tincture of galls, or kino, or catechu, or the gallic acid, or the creosote, or some other styptic, should be pushed up the nostril. Blowing some pulverulent matter through a tube into the nostril, flour, or starch, or chalk, or Armenian bole, or charcoal, sometimes answers even better. The coal of a burnt cork, pulverized, is particularly recommended by Sims of London; the dust forming a coat over the surface of the membrane, chokes up the mouths of the patulous vessels.

In very intractable cases the head may be dipped in cold water, rendered intensely so by the addition of ice, which is said to be decisive by Darwen and other authorities.

An immersion of the whole body in a cold bath has been advised, and we are not without evidence of its complete success.* The patient, however, should continue in it for some time, so as to attain the sedative effect, or otherwise the object would be defeated by the powerful reaction excited by the sudden impression of the remedy. Yet it is the more common, in such emergencies, to endeavour to effect compression, by introducing a piece of sponge, properly shaped, into the nostril. This may be pushed in by a probe, or, where the bleeding proceeds from vessels very high up, it is suggested to tie a piece of catgut to the sponge, carry it through the posterior nares by a probe, and out of the mouth, by which the sponge can be completely drawn up. But though this is generally recommended, it will, I apprehend, be found exceedingly difficult in execution, from the extreme irritation induced in the muscles of the pharynx, and is altogether so uncomfortable, that it will seldom be submitted to long enough to be of any service. I recollect that it was a remark of Mr. Abernethy in one of his lectures, that though he had often tried to do it, he had been uniformly baffled in the attempts. But, at the same time, he told us, that he had not seen a case in which he did not succeed in suppressing the hæmorrhage, by a plug exactly shaped to the cavity of the nostril, made of lint, first wetted, and wound round a probe, which may be withdrawn on the introduction of the lint, keeping the latter in for several days.

An emetic, except it be positively contra-indicated by great exhaustion, I think ought to be tried in an emergency. It is only in a single

* Philadelphia Journal of the Medical Sciences, vol. ii.

case, that of an aged and infirm man, that I have ventured on the practice,—and though it did not succeed, it was productive of no mischief. Emetics, however, on the authority of Stoll, have been used advantageously. They sometimes prove very decisive in diverting blood from the head,—and their control over the capillary system is not less established, on which views, we should be warranted in resorting to them, independently of the positive testimony I have cited, or any analogical conclusion from their efficacy in other hæmorrhages.

To evacuations of the bowels, unless constipation exists, no importance has been attached. But on every account, it seems to me, that a strong impression by an active, even drastic cathartic, promises well. The acetate of lead, and similar articles, alleged to be so useful in most other hæmorrhages, I do not think exercise here any power. But I am strongly inclined to suspect that opiates are of great value. For the last few years, I have occasionally directed them with success. It is, indeed, only a week or two ago, that I witnessed signal advantage from a dose of the Dover's powder, in a case that previously had very obstinately resisted the customary means, to which I was called in consultation with Dr. Jackson. The Dover's powder I have found to be incomparatively the best of the opiate preparations. How it operates, I pretend not to determine with any precision. It may be by producing a change in the nervous system, certain conditions of which must undoubtedly influence hæmorrhage,—though something, I think, is also ascribable to the emetic substance entering into the composition of the remedy, as without it, neither opium, nor its simple preparations, have an equally beneficial effect,—and this conjecture is rendered the more probable, from the reputation ipecacuanha, in small portions, has long had in the hæmorrhagic affections.

Having arrested the bleeding, the patient may be permitted to repose in bed, with the head and shoulders elevated. But in children, care ought to be observed, that the blood is not flowing through the posterior nares, as it occasionally does, when apparently checked, producing much inconvenience.

To do away the disposition to a recurrence, which in some instances is exceedingly inveterate, it is required to pursue a course of prophylactic measures. As a leading part of the plan, an endeavour is to be made to equalize the circulation, the balance in which is often subverted by a tendency to the head, and at the same time cautiously to recruit the tone and energies of the system. The first of these purposes, provided there be not too great debility, is met by purging, which is useful in the affections of the head generally, and particularly so in this, from torpor of the bowels usually attending it. Counter-irritation is likewise serviceable, and a blister to the nape of the neck has of itself very frequently accomplished a cure. The application, however, of vesicatories may sometimes be made to the lower extremities, acting as divellents.

As a dernier resource, mercury urged to a slight salivation is said to have been appealed to with advantage, in singularly obstinate cases, though it is not to be indiscriminately employed. Certainly, it would prove injurious in the cachectic condition, dependent especially on a scorbutic or tubercular diathesis, and, perhaps, is most or only appropriate when the attack proceeds from lesions of the abdominal viscera, as we shall presently see.

During the progress of this treatment, it may be found, though the general circulation be weak, there are manifestations of cerebral determinations, to relieve which, cups or leeches may be applied.

Thus having prepared the system, tonics, as the sulphate of quinine, alone, or with the chalybeates, or the mineral acids, and a nourishing, though temperate diet, with regulated exercise, are to be directed.

Of the idiopathic shape of the disease, I have nothing more to say. But it is also met with of a secondary nature, proceeding from metastasis, or is the effect of diseased viscera, the liver, or spleen, &c. It is plain, in the management of such cases, having suppressed the flow, attention is to be called to the primary affection, to re-establish the hæmorrhoidal or catamenial discharge, or to remove the morbid condition of the viscus affected, as the one or the other may be the remote cause of the hæmorrhage. But I shall decline now pointing out the remedies, as I should be anticipating what will hereafter become a subject of ample discussion.

